# Girls get by with a little help from their friends: gender differences in protective effects of social support for psychotic phenomena amongst poly-victimised adolescents

**DOI:** 10.1007/s00127-018-1599-6

**Published:** 2018-09-25

**Authors:** Eloise Crush, Louise Arseneault, Helen L. Fisher

**Affiliations:** King’s College London, Social, Genetic and Developmental Psychiatry Centre, Institute of Psychiatry, Psychology and Neuroscience, London, UK

**Keywords:** Psychosis, Psychotic-like experiences, Sex differences, Resilience, Victimization

## Abstract

**Purpose:**

To investigate whether social support is protective for psychotic experiences similarly among poly-victimised adolescent girls and boys.

**Methods:**

We utilised data from the Environmental Risk (E-Risk) Longitudinal Twin Study, a nationally-representative sample of 2232 UK-born twins. Participants were privately interviewed at age 18 about victimisation, psychotic experiences, and social support during adolescence.

**Results:**

Perceived social support (overall and from friends) was found to be protective against psychotic experiences amongst poly-victimised adolescent girls, but not boys. Though boys were similarly protected by family support.

**Conclusions:**

Social support-focused interventions targeting psychotic phenomena amongst poly-victimised adolescents may be more effective for girls.

**Electronic supplementary material:**

The online version of this article (10.1007/s00127-018-1599-6) contains supplementary material, which is available to authorized users.

## Introduction

A lack of social support has been associated with the emergence of psychotic symptoms (e.g., hearing voices or feeling very paranoid) in the general population [1, 2] and full-blown psychotic disorders [3]. Conversely, increased levels of perceived social support have been linked to an absence of psychotic experiences amongst adolescents at high risk due to exposure to multiple forms of victimisation (poly-victimised) [4]. Research has suggested that social support may buffer the effects of stress [5–8], improve self-esteem [9–11], and reduce feelings of loneliness [12, 13], which may all protect against psychotic phenomena.

Studies have commonly reported that social support is more strongly associated with an absence of psychopathology amongst adolescent girls [8, 14–18]. One study found social support buffered against psychotic disorders specifically amongst women exposed to physical abuse in childhood [19]. The current study aims to extend this work by exploring whether the protective effects of perceived social support vary by gender in relation to sub-clinical psychotic experiences amongst poly-victimised adolescents in the general population. In this study, we focus on adolescents’ perceptions of the amount of social support they receive from friends, family and significant others, and thus capture both the perceived availability and also functional aspects of social support [20].

## Methods

### Study cohort

Participants were members of the Environmental Risk (E-Risk) Longitudinal Twin Study, a nationally-representative birth cohort of 2232 twin children born in England and Wales in 1994–1995. Full details about the sample are reported elsewhere [21], and in the Supplementary Materials. Briefly, the E-Risk sample was constructed in 1999–2000, when 1116 families with same-sex 5-year-old twins (93% of those eligible) participated in home-visit assessments. Families were recruited to represent the UK population of families with newborns in the 1990s, based on residential location throughout England and Wales and mothers’ age. The sample comprised 56% monozygotic and 44% dizygotic twin pairs, and sex was evenly distributed within zygosity (49% male). Follow-up home-visits were conducted when children were aged 7 (98% participation rate), 10 (96%), 12 (96%), and 18 years (93%).

### Adolescent poly-victimisation

At age 18, participants were interviewed about exposure to seven different forms of victimisation (crime, peer/sibling, internet/mobile phone, sexual, family violence, maltreatment and neglect) between 12 and 18 years using the Juvenile Victimization Questionnaire, 2nd revision (JVQ-R2) [22] adapted as a clinical interview [23]. The worst experience (according to the participant) for each victimisation type was rated by trained coders using a six-point scale: 0 = not exposed, then 1–5 for increasing levels of severity (see Supplementary Materials). The adolescent poly-victimisation variable was derived by summing all victimisation experiences that received a code of ‘4’ or ‘5’ (i.e., severe exposure). Due to small numbers in some of the groups, we collapsed this variable into ‘0’ not victimised (64.6%), ‘1’ experienced 1 type of severe victimisation (19.2%), and ‘2’ poly-victimised (16.2%, experienced 2 or more types of severe victimisation).

### Adolescent psychotic phenomena

At age 18, each participant was privately interviewed about 13 psychotic experiences occurring since age 12. Seven items pertained to delusions and hallucinations and this interview has been described in detail previously [24] and in the Supplementary Materials. Six items pertained to unusual experiences which drew on item pools since formalised in prodromal psychosis instruments including the PRIME-screen and SIPS [25]. All 13 items were summed to create a psychotic experiences scale (range 0–18, M 1.19, SD 2.58). Just over 30% of participants reported at least 1 psychotic experience between ages 12 and 18 (*N* = 623, 30.2%).

### Social support

Social support was assessed at age 18 using the Multidimensional Scale of Perceived Social Support (MSPSS), which assesses participants’ access to supportive relationships with family, friends and significant others [26]. Participants rated the 12 items as “not true” (0), “somewhat true” (1), or “very true” (2). We summed scores to produce an overall social support scale with higher scores reflecting greater social support (internal consistency: *α* = 0.88). In addition, each of the three sub-scales was utilised separately to examine whether social support from either family, friends or significant others was found to be specifically protective.

### Potential confounders

Family socioeconomic status (SES) was measured when participants were aged 5 via a composite of parental income (total household), education (highest for mother/father), and occupation (highest for mother/father) [27], and was categorised into tertiles (i.e., low-, medium-, and high-SES). Mothers reported on family history of psychiatric disorders [28] in private interviews when participants were aged 12, which was converted to a proportion (0–1.0) of family members with a history of psychiatric disorder [29]. Childhood psychotic symptoms pertaining to 7 delusions and hallucinations were measured when participants were aged 12 during private interviews and verified by clinicians [24]. A total of 5.9% of the sample reported experiencing at least one definite psychotic symptom at age 12 (*N* = 125). A variable was also created for the presence vs. absence of any childhood mental health problems to capture children who met criteria for extreme anxiety, clinically-relevant depression symptoms, attention deficit hyperactivity disorder (ADHD), or conduct disorder by age 12 (see Supplementary Materials).

### Statistical analysis

We used logistic regression to test the association between (1) poly-victimisation and psychotic experiences at age 18, and (2) social support and age-18 psychotic experiences among all participants exposed to poly-victimisation (*N* = 334) and then separately for boys and girls. We tested for gender differences in the association between social support and psychotic experiences by including a ‘gender × social support’ interaction term in the regression analysis. All of these analyses were adjusted for family SES, family psychiatric history, age-12 psychotic symptoms, and childhood mental health problems. Analyses were conducted in STATA 11.2 (Stata-Corp, College Station, TX, USA). Because each study family contains two children, all statistical analyses were corrected conservatively for the non-independence of twin observations using tests based on the Huber/White variance estimator [30].

## Results

Poly-victimisation was associated with an increased likelihood of psychotic experiences at age 18 after controlling for confounders (OR 3.81; 95% CI 2.92–4.97). There were no differences in this association between boys and girls (interaction OR 0.76; 95% CI 0.44–1.30).

Higher perceived levels of social support were found to be associated with a decreased likelihood of adolescent psychotic experiences amongst those exposed to poly-victimisation (OR 0.93; 95% CI 0.88–0.98). Next, we considered whether social support was protective for both boys and girls exposed to poly-victimisation (Table 1). We found a statistically significant interaction between gender and social support (interaction OR 0.88; 95% CI 0.79–0.98), such that total social support was only protective amongst girls (OR 0.88, 95% CI 0.82–0.94) but not boys (OR 0.99, 95% CI 0.92–1.07). None of the social support sub-types were significantly protective for adolescent boys, albeit there was a strong trend for family social support being protective (OR 0.83, 95% CI 0.66–1.04). Among the social support sub-types, gender differences were only statistically significant for the association between social support from friends and an absence of psychotic experiences (Table 1), with the protective effect evident for girls.

**Table 1:** Associations between social support and age-18 psychotic experiences amongst adolescents exposed to poly-victimisation, split by gender

Social support subscale	Boys *N* = 140				Girls *N* = 192				Sex differences
	No psychotic experiences *N* = 50 *n* (%)	Psychotic experiences *N* = 90 *n* (%)	Unadjusted OR (95% CI)	Adjusted OR^a^ (95% CI)	No psychotic experiences *N* = 84 *n* (%)	Psychotic experiences *N* = 108 *n* (%)	Unadjusted OR (95% CI)	Adjusted OR^a^ (95% CI)	Interaction OR^a^ (95% CI)
Total	18.6 (6.2)	18.4 (4.9)	0.99 (0.92–1.07)	0.99 (0.92–1.07)	21.0 (3.9)	17.8 (5.7)	**0.87 (0.82–0.93)**	**0.88 (0.82–0.94)**	**0.88 (0.79–0.98)**
Family	6.6 (2.2)	5.7 (2.4)	0.84 (0.68–1.03)	0.83 (0.66–1.04)	6.7 (2.2)	5.5 (2.7)	**0.81 (0.71–0.93)**	**0.83 (0.72–0.96)**	1.00 (0.75–1.32)
Friends	5.6 (2.6)	5.9 (2.4)	1.06 (0.92–1.21)	1.06 (0.92–1.22)	6.9 (1.8)	5.4 (2.7)	**0.75 (0.65–0.87)**	**0.77 (0.66–0.89)**	**0.72 (0.59–0.89)**
Significant others	6.4 (2.5)	6.7 (2.0)	1.06 (0.90–1.24)	1.07 (0.89–1.28)	7.4 (1.5)	6.9 (2.0)	0.86 (0.72–1.03)	0.86 (0.71–1.03)	0.79 (0.61–1.02)

Bold text indicates *p* < 0.05

*OR* odds ratio, *CI* confidence interval

^a^Controlling for family socioeconomic status, family psychiatric history, age-12 psychotic symptoms, other mental health problems at age 12, and the non-independence of twin observations

## Discussion

To our knowledge, this is the first study to investigate gender differences in the buffering effect of social support for psychotic experiences amongst poly-victimised adolescents in the general population. Broadly, our results suggest perceived social support is more protective amongst adolescent girls exposed to poly-victimisation, than amongst boys. Evidence was found for total perceived social support, and support from family and friends, to be protective in relation to psychotic experiences among girls exposed to multiple forms of victimisation. Amongst boys there was a strong trend for family support to be protective but the association failed to meet conventional levels of statistical significance.

Social support has been found to improve self-esteem particularly amongst girls [9] and, therefore, it is plausible that the protective nature of social support from friends and family for adolescent girls exposed to poly-victimisation in this sample can be explained in part due to improvements in self-esteem. Indeed, low self-esteem has been found to be predictive of psychotic phenomena in non-clinical populations [31] and has been shown to mediate associations between victimisation and adolescent psychotic experiences [32]. Relatedly, research has found girls rely on social support as a coping strategy more often than boys [33, 34], which may be particularly important for buffering stress related to poly-victimisation exposure.

Limitations should be considered. First, our cohort has a small number of adolescents exposed to poly-victimisation (*N* = 332) which may have limited statistical power to detect interactions between gender and perceived social support. In particular, the sample size may have prevented the identification of a significant effect of support from family being protective for boys. In addition, our psychotic experiences measure was self-report and, therefore, may have captured genuine experiences. Finally, our social support and psychotic experiences measures were both collected at age 18 and, therefore, it is not possible to infer the directionality of the association between them.

If replicated in larger cohorts, our findings have potential implications for interventions to prevent psychotic phenomena developing amongst adolescents exposed to poly-victimisation. Whilst social support represents a practically relevant and promising area for intervention efforts, it is possible that such interventions may be more relevant to girls and alternative strategies (or those focused on improving family support) might be more effective for boys.

## Electronic supplementary material

Below is the link to the electronic supplementary material.

Supplementary material 1 (DOC 56 KB)

## References

[1] Wiles NJ, Zammit SG, Bebbington P, Singleton N, Meltzer H, Lewis G (2006). Self-reported psychotic symptoms in the general population: results from the longitudinal study of the British National Psychiatric Morbidity Survey. Br J Psychiatry.

[2] Freeman D, McManus S, Brugha T, Meltzer H, Jenkins R, Bebbington P (2011). Concomitants of paranoia in the general population. Psychol Med.

[3] Gayer-Anderson C, Morgan C (2013). Social networks, support and early psychosis: a systematic review. Epidemiol Psychiatr Sci.

[4] Crush E, Arseneault L, Moffitt TE, Danese A, Caspi A, Jaffee J, Matthews T, Fisher HL (2018). Protective factors for psychotic experiences amongst adolescents exposed to multiple forms of victimization. J Psychiatr Res.

[5] Cohen S, Wills TA (1985). Stress, social support, and the buffering hypothesis. Psychol Bull.

[6] Olstad R, Sexton H, Søgaard AJ (2001). The Finnmark study. A prospective population study of the social support buffer hypothesis, specific stressors and mental distress. Soc Psychiatry Psychiatr Epidemiol.

[7] Prinstein MJ, Guyer AE (2005). Peer victimization, cue interpretation, and internalizing symptoms: preliminary concurrent and longitudinal findings for children and adolescents. J Clin Child Adolesc Psychol.

[8] Stadler C, Feifel J, Rohrmann S, Vermeiren R, Poustka F (2010). Peer-victimization and mental health problems in adolescents: are parental and school support protective?. Child Psychiatry Hum Dev.

[9] Bolognini M, Plancherel B, Bettschart W, Halfon O (1996). Self-esteem and mental health in early adolescence: development and gender differences. J Adolesc.

[10] Smith B, Fowler DG, Freeman D, Bebbington P, Bashforth H, Garety P, Dunn G, Kuipers E (2006). Emotion and psychosis: links between depression, self-esteem, negative schematic beliefs and delusions and hallucinations. Schizophr Res.

[11] Turner HA, Shattuck A, Finkelhor D, Hamby S (2015). Effects of poly-victimization on adolescent social support, self-concept, and psychological distress. J Interpers Violence.

[12] Sündermann O, Onwumere J, Kane F, Morgan C, Kuipers E (2014). Social networks and support in first-episode psychosis: exploring the role of loneliness and anxiety. Soc Psychiatry Psychiatr Epidemiol.

[13] Lim MH, Gleeson JFM, Alvarez-Jimenez M, Penn DL (2018). Loneliness in psychosis: a systematic review. Soc Psychiatry Psychiatr Epidemiol.

[14] Rubin C, Rubenstein J, Stechler G, Heeren T, Halton A, Housman D, Kasten L (1992). Depressive affect in “normal” adolescents: relationship to life stress, family and friends. Am J Orthopsychiatr Assoc.

[15] Landman-Peeters KMC, Hartman CA, Van Der Pompe G, Den Boer JA, Minderaa RB, Ormel J (2005). Gender differences in the relation between social support, problems in parent-offspring communication, and depression and anxiety. Soc Sci Med.

[16] Rueger SY, Malecki CK, Demaray MK (2010). Relationship between multiple sources of perceived social support and psychological and academic adjustment in early adolescence: comparisons across gender. J Youth Adolesc.

[17] Quiroga A, López-Rodríguez L, Willis GB (2017). Parental support buffering the effect of violence on adolescents’ depression: gender differences. J Interpers Violence.

[18] Van Droogenbroeck F, Spruyt B, Keppens G (2018). Gender differences in mental health problems among adolescents and the role of social support: results from the Belgian health interview surveys 2008 and 2013. BMC Psychiatry.

[19] Gayer-Anderson C, Fisher HL, Fearon P, Hutchinson G, Morgan K, Dazzan P, Boydell J, Doody GA, Jones PB, Murray RM, Craig TK, Morgan C (2015). Gender differences in the association between childhood physical and sexual abuse, social support and psychosis. Soc Psychiatry Psychiatr Epidemiol.

[20] Valtorta NK, Kanaan M, Gilbody S, Hanratty B (2016). Loneliness, social isolation and social relationships: what are we measuring? A novel framework for classifying and comparing tools. BMJ Open.

[21] Moffitt TE, The E-Risk Team (2002). Teen-aged mothers in contemporary Britain. J Child Psychol Psychiatry.

[22] Finkelhor D, Hamby SL, Turner H, Ormod R (2011). The Juvenile Victimization Questionnaire: 2nd revision (JVQ-R2).

[23] Fisher HL, Caspi A, Moffitt TE, Wertz J, Gray R, Newbury J, Ambler A, Zavos H, Danese A, Mill J, Odgers C, Pariante C, Wong CCY, Arseneault L (2015). Measuring adolescents’ exposure to victimization: The Environmental Risk (E-Risk) Longitudinal Twin Study. Dev Psychopathol.

[24] Polanczyk G, Moffitt TE, Arseneault L, Cannon M, Ambler A, Keefe RSE, Houts R, Odgers CL, Caspi A (2010). Etiological and clinical features of childhood psychotic symptoms. Arch Gen Psychiatry.

[25] Loewy RL, Pearson R, Vinogradov S, Bearden CE, Cannon TD (2011). Psychosis risk screening with the Prodromal Questionnaire-brief version (PQ-B). Schizophr Res.

[26] Zimet G, Powell S, Farley G, Werkman S, Berkoff K (1988). Psychometric characteristics of the multidimensional scale of perceived social support. J Personal Assess.

[27] Trzesniewski KH, Moffitt TE, Caspi A, Taylor A, Maughan B (2006). Revisiting the association between reading achievement and antisocial behavior: new evidence of an environmental explanation from a twin study. Child Dev.

[28] Weissman MM (2000). Brief screening for family psychiatric history: the family history screen. Arch Gen Psychiatry.

[29] Milne BJ, Caspi A, Crump R, Poulton R, Rutter M, Sears MR, Moffitt TE (2008). How should we construct psychiatric family history scores? A comparison of alternative approaches from the Dunedin Family Health History Study. Psychol Med.

[30] Williams RL (2000). A note on robust variance estimation for cluster-correlated data. Biometrics.

[31] Krabbendam L, Janssen I, Bak M, Bijl RV, de Graaf R, van Os J (2002). Neuroticism and low self-esteem as risk factors for psychosis. Soc Psychiatry Psychiatr Epidemiol.

[32] Fisher HL, Schreier A, Zammit S, Maughan B, Munafo M, Lewis G, Wolke D (2013). Pathways between childhood victimization and psychosis-like symptoms in the ALSPAC birth cohort. Schizophr Bull.

[33] Eschenbeck H, Kohlmann C-W, Lohaus A (2007). Gender differences in coping strategies in children and adolescents. J Individ Differ.

[34] Rose AJ, Rudolph KD (2006). A review of sex differences in peer relationship processes: potential trade-offs for the emotional and behavioral development of boys and girls. Psychol Bull.

