# Correction to: Girls get by with a little help from their friends: gender differences in protective effects of social support for psychotic phenomena amongst poly-victimised adolescents

**DOI:** 10.1007/s00127-018-1620-0

**Published:** 2018-11-02

**Authors:** Eloise Crush, Louise Arseneault, Helen L. Fisher

**Affiliations:** King’s College London, Social, Genetic and Developmental Psychiatry Centre, Institute of Psychiatry, Psychology and Neuroscience, London, UK

## Correction to: Social Psychiatry and Psychiatric Epidemiology 10.1007/s00127-018-1599-6

The article Girls get by with a little help from their friends: gender differences in protective effects of social support for psychotic phenomena amongst poly-victimised adolescents, written by Eloise Crush, Louise Arseneault and Helen L. Fisher, was originally published electronically on the publisher’s internet portal (currently SpringerLink) on 25 September 2018 without open access.

With the author(s)’ decision to opt for Open Choice the copyright of the article changed on 2 September 2018 to © The Author(s) 2018 and the article is forthwith distributed under the terms of the Creative Commons Attribution 4.0 International License (http://creativecommons.org/licenses/by/4.0/), which permits use, duplication, adaptation, distribution and reproduction in any medium or format, as long as you give appropriate credit to the original author(s) and the source, provide a link to the Creative Commons license and indicate if changes were made.

